# Laparoscopic Microwave Ablation and Salvage Liver Transplantation in Patients with Hepatocellular Carcinoma

**DOI:** 10.3390/cancers17132248

**Published:** 2025-07-04

**Authors:** Alessandro Vitale, Marco Brolese, Ilaria Govoni, Chiara Naldini, Nicola Canitano, Enrico Gringeri, Francesco D’Amico, Domenico Bassi, Francesco Enrico D’Amico, Jacopo Lanari, Alessandro Furlanetto, Virginia Padoan, Daniel Salinas, Umberto Cillo

**Affiliations:** 1 Unità di Chirurgia Epatobiliopancreatica e Trapianto Epatico, Azienda Ospedaliera-Università di Padova, 35128 Padova, Italy; alessandro.vitale@unipd.it (A.V.); chiara.naldini@aopd.veneto.it (C.N.); nicola.canitano@aopd.veneto.it (N.C.); enrico.gringeri@unipd.it (E.G.); domenico.bassi@aopd.veneto.it (D.B.); francescoenrico.damico@unipd.it (F.E.D.); jacopo.lanari@unipd.it (J.L.); alessandro.furlanetto@unipd.it (A.F.); virginia.padoan@studenti.unipd.it (V.P.); cillo@unipd.it (U.C.); 2 Dipartimento di Medicina di Precisione e Rigenerativa e Area Jonica (DiMePRe-J), Bari University, 70121 Bari, Italy; francesco.damico1@uniba.it; 3 Medicina Generale, ULSS3 Ospedale dell’Angelo, 30174 Venezia, Italy; daniel.salinas@aulss3.veneto.it

**Keywords:** hepatocellular carcinoma, salvage liver transplantation, laparoscopic microwave ablation, graft shortage, hcc recurrence

## Abstract

**Background/Objectives**: Salvage liver transplantation (SLT) is a well-established option for hepatocellular carcinoma (HCC) recurrence after liver resection. Laparoscopic microwave ablation (L-MWA) represents another curative strategy for early-stage HCC. However, its role within this therapeutic framework remains unexplored. **Methods**: Between 2014 and 2023, we treated 1341 patients with HCC using L-MWA. From this cohort, patients with Child-Pugh class A liver function, HCC within the Milan criteria, no contraindications to transplantation, and ≥12 months of follow-up were selected. SLT failure was defined as non-transplantable recurrence or death, resulting in the loss of a potentially curative therapeutic opportunity. The primary endpoint was overall survival (OS); secondary endpoints included predictors of survival and SLT failure. **Results:** A total of 341 patients met the inclusion criteria. Five-year OS was 62%. Independent predictors of poorer survival included the presence of cardiac disease or oesophageal varices, a Child-Pugh score of 6, tumour size, and elevated alpha-fetoprotein (AFP) levels. Treatment was successful in 255 patients (74.8%): 102 (29.9%) underwent SLT, 67 (19.6%) received alternative therapies, and 93 (27.3%) remained recurrence-free. Treatment failure occurred in 86 patients (25.2%) due to non-transplantable recurrence or death. Independent predictors of failure included older age, non-HBV aetiology, and elevated AFP levels. Five-year OS rates were 79% in the success group and 22% in the failure group (*p* < 0.001). **Conclusions**: A combined L-MWA and SLT strategy is safe and effective, yielding a 62% 5-year OS rate. This approach supports more efficient graft use with a consequent increase in the population transplant benefit. Improved selection may further reduce failure rates.

## Simple Summary

This study investigates the effectiveness of a combined strategy involving laparoscopic microwave ablation (L-MWA) as a first-line curative treatment for hepatocellular carcinoma (HCC) and salvage liver transplantation (SLT) in cases of HCC recurrence. Given the limited availability of donor organs, the demand for effective alternatives is increasing. While L-MWA has shown promising results in treating small tumours, this research is the first to assess the feasibility and efficacy of incorporating L-MWA into a “salvage liver transplantation” approach. Beyond evaluating patient outcomes, the study aims to identify predictors of survival and treatment failure. The findings could improve the management of HCC by optimising the use of scarce organ resources and enhancing the transplant benefit for the population.

## 1. Introduction

Liver transplantation (LT) serves as a benchmark treatment for patients with hepatocellular carcinoma (HCC) in the context of liver cirrhosis, effectively addressing both the malignancy and the underlying liver disease.

Despite efforts to expand the donor pool, including the use of marginal grafts, donation after circulatory death (DCD), machine perfusion, and living donor liver transplantation (LDLT), a significant disparity between organ availability and demand persists [1,2]. Notably, over the past two decades, advances in downstaging and conversion therapies have broadened the indications for liver transplantation in HCC [3,4,5] and have also introduced transplant eligibility in selected cases of colorectal liver metastases (CRLM) [6], cholangiocarcinoma [7], and neuroendocrine tumour (NET) metastases [8]. This has necessitated the development of prioritisation strategies based on “transplant benefit,” in which LT is primarily reserved for patients lacking effective curative alternatives [9]. Conversely, in patients with HCC who are eligible for potentially curative treatments such as liver resection or ablation, transplantation should be considered only in a salvage setting.

In the late 1990s, to address organ shortages and waitlist drop-out, liver resection (LR) was increasingly used as the first-line therapy for LT-eligible HCC patients, with LT reserved for recurrence or liver failure—a strategy later formalised by Majno et al. as “salvage liver transplantation” (SLT) [10,11]. De Haas et al. subsequently defined SLT “failure” as untreatable recurrence or drop-out [12]. While SLT and primary LT (PLT) offer comparable overall survival rates in selected cases, SLT failure remains a frequent occurrence [13,14]. A major challenge is optimally selecting patients—balancing the benefits of LT with the curative potential of alternatives while minimising unnecessary graft use. Markers such as elevated AFP, large tumours, and multinodularity assist in risk stratification [15], but some patients remain poorly characterised. Percutaneous microwave ablation (P-MWA) has gained recognition as a curative option for small HCCs and has been explored as a first-line treatment within an SLT strategy [16,17]. However, its use is limited in cases of ascites, multifocal disease, or anatomically challenging lesions [18].

In this context, laparoscopic microwave ablation (L-MWA) has emerged as a promising alternative for patients who are unsuitable for LR or P-MWA, following the “Therapeutic Hierarchy” concept [19]. At our institution, L-MWA has been successfully integrated into clinical practice, achieving excellent oncologic outcomes: previous series have reported local tumour control rates exceeding 75% and encouraging overall survival, with 1-year, 3-year, and 5-year OS rates of 81.9%, 54.9%, and 35.9%, respectively [20,21].

Despite the established role of LR and percutaneous ablation within SLT strategies, no studies to date have evaluated the use of L-MWA as a first-line curative-intent treatment in this setting.

This study aimed to assess the feasibility and oncologic effectiveness of L-MWA within an SLT strategy through an extensive retrospective analysis, while also focusing on identifying predictors of overall survival (OS), evaluating the incidence of SLT strategy failure, and determining factors associated with SLT failure.

## 2. Materials and Methods

### 2.1. Patient Population and Data Collection

This retrospective, single-centre study analysed patients who underwent L-MWA for HCC at the Liver Transplantation Unit of Padua University Hospital, Italy, between January 2014 and December 2023. Eligible patients were adults aged 18 years or older with a diagnosis of HCC according to the European Association for the Study of the Liver (EASL) guidelines [22].

Patients were excluded if they were older than 75, had recurrent HCC, or presented with an Eastern Cooperative Oncology Group (ECOG) performance status greater than 2 [23]. Further exclusion criteria included Child-Pugh class B or C cirrhosis [24], tumours beyond the Milan Criteria [25], unsuitability for LT or curative intent L-MWA (due to age, comorbidities, or tumour characteristics), and a follow-up duration of less than one year.

Baseline characteristics collected included age, sex, body mass index (BMI), presence of comorbidities, presence and aetiology of liver cirrhosis (HCV infection, HBV infection, alcohol-related liver disease, metabolic-associated steatotic liver disease [MASLD], or mixed aetiologies), serum alpha-fetoprotein (AFP) levels (ng/mL), presence of cirrhosis-related complications, Albumin–Bilirubin (ALBI) grade, Model for End-stage Liver Disease (MELD) score [26] and tumour burden characteristics (number and size of HCC nodules, and Barcelona Clinic Liver Cancer [BCLC] stage [27]).

### 2.2. Surgical Procedure and Follow-Up

The indications for and the technical details of laparoscopic MWA have been previously described [20] and are summarised in Appendix A. Briefly, a laparoscopic approach was selected when a percutaneous strategy was deemed not technically feasible. The procedure involved the insertion of a 14-G water-cooled coaxial antenna directly into the tumour under real-time ultrasound guidance. Ablation was performed using a 2.45 GHz microwave generator (AMICA GEN; HS Hospital Service SpA, Aprilia, Italy) with a median energy delivery of 60 W.

Short-term perioperative outcomes assessed included operative time, length of hospital stay (LOS), postoperative complications classified according to the Clavien–Dindo system [28], and 30-day mortality.

Postoperative follow-up included routine clinical assessments, monitoring of AFP levels, and contrast-enhanced computed tomography (CT) of the thorax and abdomen. This was performed 30 days after the procedure, then every 3 months during the first 2 years, and every 6 months thereafter for up to 5 years.

In case of recurrence eligible for further treatment, patients were managed according to institutional protocols based on the “Therapeutic Hierarchy” and “Converse Therapeutic Hierarchy” frameworks [19,29]. Treatment options included LT, repeat L-MWA, liver resection (LR), transarterial chemoembolisation (TACE), or systemic therapy (ST). Patients with recurrence who were not amenable to any treatment received the best supportive care.

### 2.3. Study Design

**Figure 1:**
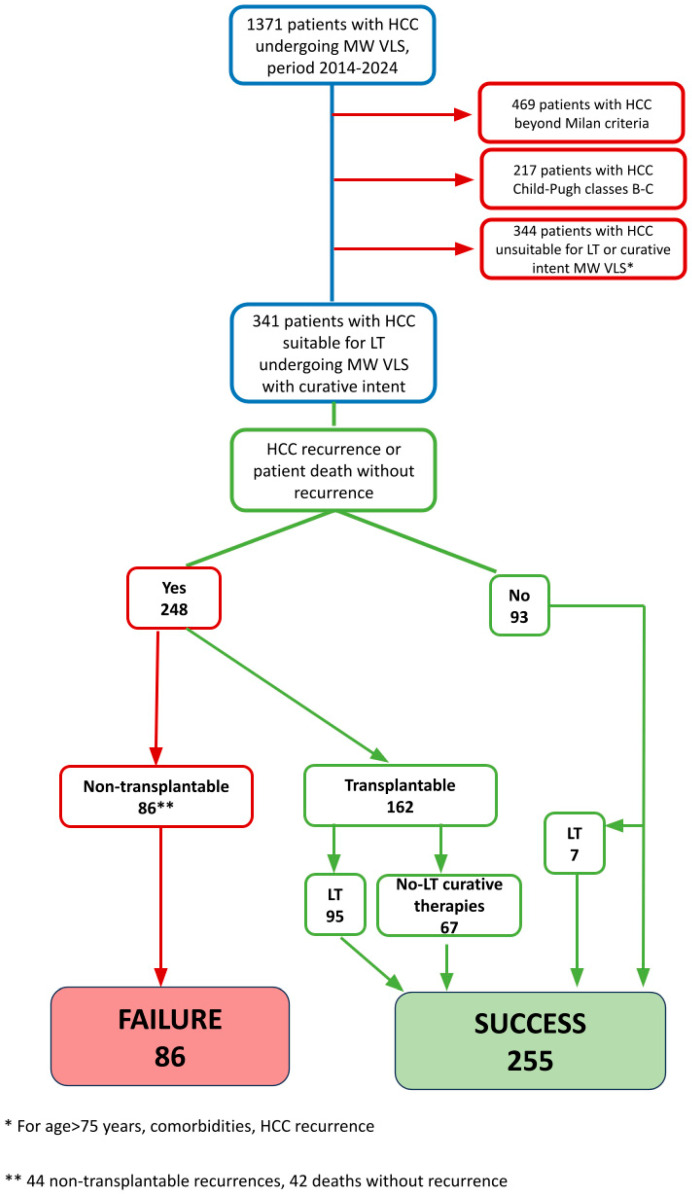
Flow diagram of patient selection and outcome classification. HCC: hepatocellular carcinoma; L-MWA: laparoscopic microwave ablation; LT: liver transplantation.

A total of 1371 patients with HCC who underwent L-MWA between January 2014 and December 2023 were initially considered. Of these, 469 patients with HCC beyond the Milan criteria, 217 patients with Child-Pugh class B or C cirrhosis, and 344 patients deemed unsuitable for LT or curative intent L-MWA (due to age >75 years, significant comorbidities, or HCC recurrence) were excluded. After applying these criteria, 341 patients with HCC suitable for LT and undergoing L-MWA with curative intent were included in the final analysis. The study design, including patient selection and outcome definitions, is illustrated in Figure 1.

The primary endpoint of the study was overall survival (OS), defined as the time from the initial L-MWA procedure to death from any cause or the last follow-up. Secondary endpoints included identifying predictors of OS, assessing the incidence of SLT strategy failure, and identifying predictors of SLT strategy failure.

The success of the SLT strategy was defined as either the absence of HCC recurrence during at least one year of follow-up after L-MWA or the successful completion of LT. Failure of the SLT strategy was defined as the occurrence of recurrence not amenable to curative treatment or patient death from non-oncologic causes before LT could be performed.

### 2.4. Statistical Analysis

Categorical variables were summarised as absolute frequencies and percentages, while continuous variables were reported as medians with interquartile ranges (IQR). Comparisons between categorical variables were conducted using Pearson’s chi-squared test or Fisher’s exact test, as appropriate. Continuous variables were assessed using the Kruskal–Wallis rank sum test. Follow-up duration and survival times were presented as medians with corresponding IQRs.

OS was estimated using the Kaplan–Meier method, and differences between survival curves were assessed with the log-rank test. Univariable and multivariable Cox proportional hazards regression models were used to identify prognostic factors for OS. Univariable and multivariable logistic regression analyses were performed to explore predictors of SLT strategy failure, with results reported as odds ratios (OR) and 95% confidence intervals (CI).

Variables with a *p*-value < 0.1 in univariable analysis or those judged clinically relevant were included in the multivariable models. A two-sided *p*-value < 0.05 was considered statistically significant. All statistical analyses were performed using JMP software, Version 18.2.0 (SAS Institute, Cary, NC, USA 2024–2025) and StataNow/SE 19.5 (Copyright 1985-2025 StataCorp LLC, College Station, TX, USA).

## 3. Results

### 3.1. Patient Characteristics and Short-Term Perioperative Outcomes

A total of 341 patients with HCC suitable for LT and undergoing L-MWA with curative intent were included. Baseline characteristics are summarised in Table 1.

The median age was 64 (IQR 57–69), and 57 patients (16.7%) were female. Liver cirrhosis was present in 94.1% of cases, most commonly related to HCV infection (41.3%), alcohol-related liver disease (32.6%), HBV infection (17.3%), and MAFLD (17.3%). Diabetes mellitus was reported in 34.6%, and cardiopulmonary comorbidities in 13.2%, with a median Charlson Comorbidity Index of 4 (IQR 3–5). Signs of portal hypertension were common, with oesophageal varices detected in 39.3%, ascites in 11.7%, and hepatic encephalopathy in 3.8%. The median MELD score was 6 (IQR 6–9), and 71.3% of patients had an ALBI grade ≥2.

The median total tumour diameter was 27 mm (IQR 20–35), with the largest lesion measuring a median of 22 mm (IQR 18–29). A single HCC nodule was observed in 68.3% of patients, and 70.1% were classified as BCLC stage A. AFP levels were <20 ng/mL in 72.4%, while 11.5% had AFP >100 ng/mL.

The median operative time was 75 min (IQR, 60–100), with a median hospital stay of 2 days (IQR, 2–3). Overall, 84% of patients (*n* = 286) had no postoperative complications. Among those who did, complications were classified as Clavien–Dindo grade I in 40 patients (11.7%), grade II in 14 patients (4.1%), and grade IIIb in one patient (0.3%). The latter required reintervention for hemoperitoneum. No postoperative mortality was recorded.

### 3.2. Success of the SLT Strategy

During follow-up after L-MWA, 248 patients (72.7%) experienced either HCC recurrence (*n* = 206, 60.4%) or death without recurrence (*n* = 42, 12.3%), while 93 patients (27.3%) remained event-free. Among the 248 patients who developed an event, 162 (65.3%) remained eligible for LT, whereas 86 patients (34.7%) were deemed non-transplantable. Non-transplantability was attributed to non-transplantable HCC recurrence (*n* = 45, 52.3%) or death from non-oncologic causes without recurrence (*n* = 42, 48.8%).

Among the 162 patients with HCC recurrence who remained eligible for transplantation, 95 patients (58.6%) ultimately underwent LT, while 67 patients (41.4%) received alternative curative therapies, including LR (*n* = 9) or repeat L-MWA (*n* = 58). In addition, seven patients (2.0% of the total cohort) underwent LT for decompensated cirrhosis without evidence of HCC recurrence during follow-up.

Overall, the SLT strategy was successful in 255 patients (74.8%), defined as either the completion of LT (*n* = 95, 27.9%) or sustained tumour control achieved with non-transplant curative therapies (*n* = 67, 19.6%). Failure of the SLT strategy occurred in 86 patients (25.2%).

Factors associated with failure of the SLT strategy were assessed through univariable and multivariable logistic regression analyses (Table 2). In the multivariable analysis, increasing age (per 10 years) was independently associated with a higher risk of SLT failure (odds ratio [OR], 1.49; 95% CI, 1.08–2.06; *p* = 0.016). Conversely, HBV infection was identified as a protective factor against SLT failure (OR 0.42, 95% CI 0.19–0.95, *p* = 0.037). Elevated alpha-fetoprotein (AFP) levels >100 ng/mL were also associated with an increased risk of SLT failure (OR 2.14, 95% CI 1.03–4.47, *p* = 0.042).

### 3.3. Overall Survival (OS)

**Figure 2:**
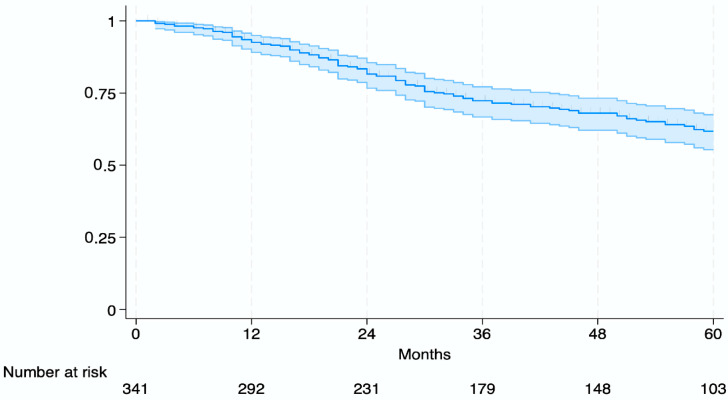
Kaplan–Meier curve of overall survival for the entire study cohort.

At a median follow-up of 58 months for survivors (IQR, 31–78 months), the 1-year, 3-year, and 5-year overall survival (OS) rates for the entire study population were 93%, 72%, and 62%, respectively (Figure 2).

**Figure 3:**
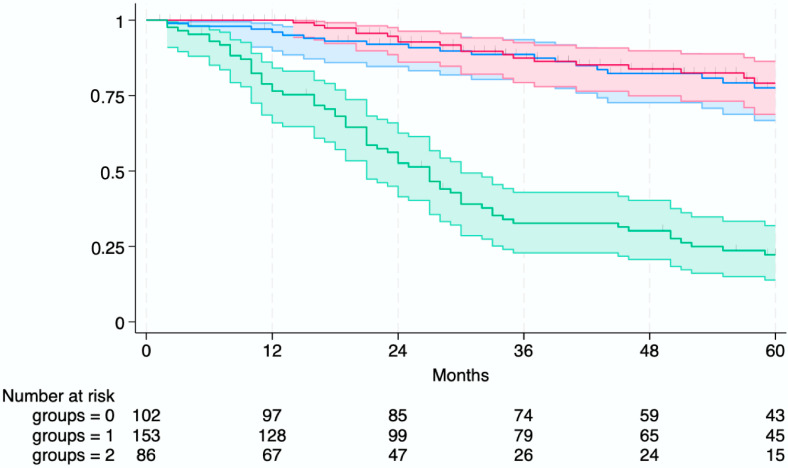
Overall survival stratified by outcome of salvage liver transplantation (SLT) strategy after laparoscopic microwave ablation (L-MWA). LT: liver transplantation; SLT: salvage liver transplantation; L-MWA: laparoscopic microwave ablation.

When stratified according to both the outcome of the SLT strategy and whether patients received an LT, a significant difference in OS was observed between the success groups and the failure group (log-rank test, *p* < 0.001) (Figure 3).

The “Success—LT” group comprised patients who achieved SLT success through LT (*n* = 102), the “Success–No LT” group included patients who achieved SLT success through non-transplant curative therapies (*n* = 153), and the “Failure” group included patients who experienced SLT failure (*n* = 86). At 5 years, the OS rates were approximately 78% for the Success-LT group, 79% for the Success-No LT group, and 22% for the Failure group (Figure 3).

The results of the univariable and multivariable Cox regression analyses for OS are summarised in Table 3. In the multivariable analysis, the presence of cardiopulmonary comorbidities (hazard ratio [HR] 1.75, 95% confidence interval [CI] 1.07–2.85, *p* = 0.025), Child-Pugh score of 6 versus 5 (HR 1.51, 95% CI 1.03–2.21, *p* = 0.036), presence of oesophageal varices (HR 1.54, 95% CI 1.05–2.24, *p* = 0.026), and largest tumour size (per cm increase; HR 1.39, 95% CI 1.10–1.75, *p* = 0.006) were independently associated with reduced OS. Additionally, alpha-fetoprotein (AFP) levels >100 ng/mL were associated with significantly worse survival (HR 2.68, 95% CI 1.62–4.44, *p* < 0.001).

## 4. Discussion

Over the years, SLT has gained a pivotal role in addressing organ shortage, optimising graft allocation while offering a potentially curative treatment for selected patients. This study represents the first attempt to evaluate L-MWA feasibility and efficacy within the SLT strategy in patients with HCC, aiming to identify predictive factors and assess oncologic outcomes. Recently, HCC treatment has evolved from rigid, stage-based algorithms [27] to the more flexible concept of “multiparametric therapeutic hierarchy,” where decisions are tailored by a multidisciplinary tumour board based on individual patient characteristics [29]. In this context, LT always represents the first treatment choice, but the limited availability of donor organs leads to a substantial risk of waitlist drop-out, reported at 15.6% among all liver transplant candidates in the 2022 U.S. Annual Liver Transplantation Report and reaching 26% in patients listed for HCC [30].

The use of machine perfusion and the introduction of a specific DCD program in our country have led to a roughly 70% increase in organ availability over the past 10 years, reaching 1.732 liver transplants performed in 2024 [31]. However, this rise in available organs has gone hand in hand with an expansion of transplant oncology criteria, leading to a larger number of patients being added to the waiting list. In this setting, as reported in a recent consensus statement [32], most organs are still allocated to patients with more advanced disease and a higher expected transplant benefit than those in our cohort, keeping the issue of waitlist drop-out highly relevant. Patients suitable for SLT are patients at low benefit by definition since they have a potentially radical alternative to LT. These findings highlight the critical need to implement effective therapeutic strategies that can reduce the risk of waitlist drop-out, serving both as a bridge to LT and, in selected cases, as a curative treatment, thereby preserving donor organs for patients with fewer curative options.

Our strategy successfully preserved donor organs by using only 102 (30%) grafts out of the 341 potentially required, whether all patients had undergone PLT. Notably, even with this selective use of LT, we achieved a 5-year OS rate of 62% in the entire cohort, which is higher than the outcomes reported in a recent meta-analysis conducted by Guerrini et al., where survival reached 53.9% after SLT following LR and 56.5% after PLT [14]. Our result is also comparable to those reported for other established curative treatments in SLT strategy, as LR [33,34] and PMWA [16], and significantly higher compared to the approximately 33.6% 5-year OSS—or 18% in a cohort exclusively comprising BCLC B patients—reported for TACE [21,35], which remains the main alternative treatment option for these patients [36], thereby maximising resource efficiency without compromising patient outcomes. Thus, the remaining 239 grafts (70%) were made available for other individuals on the waiting list, potentially improving access and outcomes for a broader group of transplant candidates. These data underline that the transplant benefit in this setting is substantial. The concept of transplant benefit encompasses not only the absolute post-transplant survival but also critically accounts for the effectiveness of alternative treatments and patient-specific prognostic factors, such as age and tumour progression [9]. By integrating LMWA into an SLT approach, this strategy maximises transplant benefit, optimising long-term survival while potentially mitigating the burden on donor organs and the waiting list.

The 5-year OS rate reaches 79% when focusing exclusively on patients for whom the SLT strategy was successful, a rate comparable to that reported in the literature after PLT, which remains the gold standard treatment with reported 5-year OS rates of up to 75% in some studies [12]. Further subdivision of the successful SLT cohort into patients who underwent transplantation after first treatment (Success-LT group) and those who achieved durable remission with L-MWA alone without requiring transplantation (Success-No LT group) demonstrated similar 5-year OS rates of 79% and 78%, respectively. These data suggest that even after the first HCC recurrence, carefully selected patients may achieve favourable outcomes with alternative curative treatments other than LT, thereby also contributing to the preservation of donor organs.

L-MWA offers particular benefit to patients who are not eligible for LR or P-MWA, often due to critical tumour location, portal hypertension, coagulopathy, or massive ascites, and, as a result, these patients are frequently directed toward palliative treatments, such as TACE and systemic therapies, respectively [37]. In this clinical scenario, L-MWA represents an optimal therapeutic solution, effectively overcoming these limitations. Moreover, Lanari et al. showed the superiority of L-MWA over laparoscopic radiofrequency (RF) ablation in reducing recurrence rates, which is likely attributable to technical advantages, including faster tumour necrosis, larger and more predictable ablation zones, and a reduced heat sink effect [38]. However, despite growing evidence supporting its safety and oncologic efficacy, L-MWA remains underrepresented in major clinical guidelines [22,27], and it is still not routinely adopted.

A further advantage of this strategy is the low incidence of treatment-related complications. In our cohort, overall postoperative complications occurred in 16% of patients (46 out of 286), with severe complications (CD grade III or higher) reported in only 0.3% (one patient), who required reintervention for hemoperitoneum. Notably, no postoperative mortality was recorded. By comparison, literature data show that LR is associated with a substantial risk of complications: Cherqui et al. [33] reported a postoperative mortality rate of 4.5% and morbidity of 34%. In comparison, a meta-analysis indicated major morbidity rates ranging from 19% to 41% and mortality between 0% and 6% [39]. These findings underscore the clinical relevance of exploring less invasive curative treatments such as L-MWA within SLT strategies and support its consideration as a first-line treatment option in appropriate cases.

In our analysis, advanced age, elevated AFP serum levels (>100 ng/mL), and early recurrence—classic predictors of waitlist dropout [40]—were overrepresented and emerged as independent predictors of failure.

Advanced age is associated with a higher risk of treatment failure but does not independently predict 5-year mortality in our cohort. All patients considered were < 75 years, and this relatively younger age combined with preserved liver function (Child-Pugh A) likely could explain the lack of a significant impact of age on long-term survival. Nevertheless, older patients remain at increased risk of both early and non-transplantable HCC recurrence, which contributes to treatment failure [41]. This may be due to the fact that they are often treated less aggressively [42], because of higher burden of systemic comorbidities, such as cardiopulmonary diseases, which furthermore in our analysis emerged as independent predictors of mortality (HR 1.75; 95% CI: 1.07–2.85; *p* = 0.025,). These patients, due to their high risk of waitlist drop-out from non-transplantable recurrence, require prompt access to LT.

Elevated AFP is a well-established marker of aggressive tumour biology and has been consistently associated with increased recurrence risk, both after liver resection—as shown by Yao et al. [43]—and with markedly higher 5-year recurrence rates following liver transplantation [44]. At our institution, persistently elevated AFP levels ≥1000 ng/mL represent a strict exclusion criterion for transplantation. Eligibility is determined by a multidisciplinary tumour board, with decisions guided by treatment response, recurrence-free intervals, and both the reduction and sustained stability of AFP levels—closely aligning with the downstaging principles outlined in the UNOS-DS criteria [45]. Consistent with these findings, our study demonstrates that elevated serum AFP levels are significantly associated with both SLT failure (OR 2.14, 95% CI 1.03–4.47, *p* = 0.042) and poorer survival outcomes (HR 2.68, 95% CI 1.62–4.44, *p* < 0.001), reinforcing the pivotal role of tumour biology in shaping HCC treatment strategies.

The etiological landscape of HCC has evolved markedly over time. In our cohort, viral cirrhosis was identified in 201 patients (59%), reflecting a marked decline compared to previous decades, particularly in HCV-related cases (141 patients, 41%), which in the past accounted for up to 67% [20]. This reduction is largely attributable to the advent and widespread use of highly effective direct-acting antiviral (DAA) therapies. Conversely, non-viral etiologies have become increasingly prominent, with alcohol-related cirrhosis (111 patients, 33%) and metabolic-associated liver disease (59 patients, 17%) demonstrating a clear upward trend, consistent with global shifts in lifestyle and metabolic risk factors.

In our cohort, HBV-related cirrhosis was identified in 60 patients (17.6%) and, interestingly, emerged as a protective factor against treatment failure. HCC arising in the context of HBV infection typically develops in younger patients, earlier than those related to other aetiologies (i.e., HCV, alcoholic), and often occurs in livers with limited parenchymal impairment and relatively preserved function [46]. These findings suggest that this subgroup of patients may be particularly well-suited for SLT strategy, given their lower risk of failure and the absence of a significantly increased risk of waitlist mortality. Notably, this association has not been previously described in the literature and, therefore, warrants further investigation through prospective studies to validate its scientific robustness and clinical implications.

Patient-related factors also significantly influenced prognosis. In our Cox regression analysis, both Child-Pugh A6 versus A5 (HR 1.51; 95% CI: 1.03–2.21; *p* = 0.036) and the presence of oesophageal varices (HR 1.54; 95% CI: 1.05–2.24; *p* = 0.026) emerged as independent predictors of mortality, despite not being significantly associated with SLT failure. These variables, typically reflecting more advanced cirrhosis rather than tumour aggressiveness, likely impact prognosis by indicating progressive liver dysfunction rather than an increased risk of non-transplantable recurrence. As mortality is the main driver of SLT failure in these cases, such patients might benefit from early prioritisation for primary liver transplantation, when feasible.

The concept of tumour burden encompasses both the number and size of tumour nodules; however, in our analysis, only increased nodule size was found to be significantly associated with worse outcomes (HR 1.39; 95% CI: 1.10–1.75; *p* =0.006). Consistent with the reported efficacy of L-MWA relative to tumour size in the literature [47,48], our study included patients with nodules measuring ≤5 cm; however, the poorer outcomes observed in patients with larger nodules may be attributed in part to the need for multiple needle insertions during ablation, which increases procedural complexity and potentially raises the risk of residual disease or recurrence. Furthermore, larger tumours are often associated with more aggressive tumour biology, including higher rates of vascular invasion, greater presence of satellite nodules, and poorer histological grades, which may explain the higher likelihood of developing more aggressive disease and consequent unfavourable outcomes in these cases [49].

A critical limitation of the SLT strategy lies in the risk of losing access to LT for patients in whom the approach fails. In our cohort, the SLT strategy failed in 86 patients (25.2%), among whom the 5-year OS dropped markedly to 22%. Forty-four (51%) of these 86 patients experienced the onset of an untreatable recurrence, effectively losing the therapeutic opportunity of PLT. However, most of these failures occur in individuals who would likely have experienced waitlist dropout even under standard transplant protocols; in fact, these individuals initially presented typically with a low tumor burden and mild to moderate liver disease and therefore had low priority on the waiting list. Thus, in light of the unfavorable tumor biology suggested by the subsequent observed failure, it is likely that they would have experienced significant disease progression in any case, ultimately leading to dropout from the transplant waiting list. Survival rates following dropout have been reported in the literature as 51.7%, 33.1%, and 18.6% at 3, 6, and 12 months, respectively [30].

Moreover, these high-risk patients often exhibit aggressive tumour behaviour, for which unfavourable outcomes are expected even with access to PLT. In this context, L-MWA may function not only as a first-line therapy alternative to LT but also as a biological filter, offering a valuable observational window in the absence of validated tools to guide the timing of transplantation based on tumour dynamics. A key challenge in this field remains the identification of patients who really would benefit most from PLT, and our study contributes to this goal by identifying prognostic and predictive factors of SLT failure that may be decisive in guiding therapeutic choices. These findings underscore the need for additional implementation of SLT strategy in order to identify candidates who require PLT to achieve favourable outcomes and those who may still attain optimal results with a SLT strategy. Therefore, this approach offers a potentially curative strategy, or an effective bridge to LT, for carefully selected patients, while promoting a more efficient allocation of donor organs [50].

Our study presents some limitations. In particular, its retrospective, small cohort and single-centre design can introduce inherent biases and limit the generalizability of our findings. Moreover, the absence of a control group of patients undergoing PLT undoubtedly limits the strength of our conclusions. However, addressing this limitation would require the design of a prospective randomized trial, which may raise significant ethical concerns. These constraints underscore the need for future multicentre investigations to validate and expand upon our results, thereby strengthening the evidence base for the use of LMWA within the SLT strategy.

In future perspectives, loco-regional treatments such as L-MWA may play an increasingly strategic role not only for their cytoreductive efficacy but also for their potential to enhance tumour immunogenicity through antigen release [51]. This opens the door to future therapeutic combinations with immune checkpoint inhibitors, aiming to further improve disease control and long-term remission, especially in patients selected for SLT. Such integration would align with the evolving model of personalised, multimodal care in HCC treatment.

## 5. Conclusions

SLT strategy has gained a crucial role in the field of LT. In our study, we demonstrated that its combination with L-MWA is a safe and feasible option in accurately selected patients. Identifying predictors of failure within this setting is essential to optimize patient selection and ensure early prioritization on the waiting list for high-risk patients, thereby minimizing the likelihood of therapeutic failure. Prospective studies are warranted to validate our findings and further refine this strategy.

## Figures and Tables

**Table 1:** Patients’ characteristics.

Patients’ Characteristics	TOT (*n* = 341)
Females, *n* (%)	57 (16.7)
Age, years, median (IQR)	64 (57; 69)
ECOG performance status > 0, *n* (%)	45 (13.2)
Diabetes, *n* (%)	118 (34.6)
Cardiopulmonary disease, *n* (%)	45 (13.2)
CCI, median (IQR)	4 (3; 5)
BMI (Kg/m^2^), median (IQR)	27 (25; 29)
Liver Cirrhosis, *n* (%)	321 (94.1)
MASLD	59 (17.3)
HCV	141 (41.3)
HBV	60 (17.3)
ALD	111 (32.6)
Child Pugh Score 6, *n* (%)	111 (32.6)
Cirrhosis-related complications, *n* (%)	
Oesophageal varices	134 (39.3)
Ascites	40 (11.7)
Hepatic encephalopathy	13 (3.8)
Platelet count <100 × 10^9^/L	152 (44.6)
CRPH (%)	193 (56.6)
MELD Score, median (IQR)	6 (6; 9)
MELD score ≥ 10, *n* (%)	52 (15.2)
ALBI grade ≥ 2, *n* (%)	243 (71.3)
Tumour characteristics	
Total tumour diameter (mm), median (IQR)	27 (20; 35)
Largest lesion diameter, (mm), median (IQR)	22 (18; 29)
Tumour size class (mm), *n* (%)	
≤20	161 (47.2)
21–30	135 (39.6)
31–50	45 (13.2)
Oligonodular (2 or 3 nodules), *n* (%)	108 (31.7)
BCLC stage A, *n* (%)	239 (70.1)
AFP values ng/mL, *n* (%)	
≤20	247 (72.4)
21–100	55 (16.1)
>100	39 (11.5)

*n*: number; IQR: interquartile range; ECOG: Eastern Cooperative Oncology Group Performance Status; CCI: Charlson Comorbidity Index; BMI: body mass index; HCV: hepatitis C virus infection; HBV: hepatitis B virus infection; MASLD: metabolic dysfunction–associated steatotic liver disease; ALD: alcoholic liver disease; CRPH: clinically relevant portal hypertension; MELD: Model for End-stage Liver Disease; BCLC: Barcelona Clinic Liver Cancer; ALBI: Albumin–Bilirubin; AFP: alpha-fetoprotein.

**Table 2:** Logistic regression results—risk of SLT failure.

Variable	Univariable Analysis			Multivariable Analysis		
	OR	95% CI	*p*	OR	95% CI	*p*
Age (per 10 years)	1.52	1.11–2.07	0.009	1.49	1.08–2.06	0.016
Sex (male)	0.84	0.44–1.59	0.587	–	–	–
ECOG PST > 0 vs. 0	1.57	0.89–2.79	0.120	–	–	–
Diabetes	1.24	0.75–2.07	0.396	–	–	–
Cardiopulmonary comorbidity	1.24	0.62–2.49	0.544	–	–	–
CCI	1.01	0.90–1.14	0.857	–	–	–
BMI (per 10 units)	0.99	0.53–1.86	0.980	–	–	–
MASLD	0.63	0.31–1.28	0.204	–	–	–
HCV infection	1.41	0.86–2.31	0.169	–	–	–
HBV infection	0.40	0.18–0.88	0.023	0.42	0.19–0.95	0.037
ALD	1.23	0.74–2.06	0.424	–	–	–
Child score 6 vs. 5	1.41	0.85–2.35	0.184	–	–	–
Oesophageal varices	1.40	0.85–2.29	0.185	–	–	–
Ascites	0.84	0.38–1.85	0.674	–	–	–
Hepatic encephalopathy	1.91	0.61–5.99	0.270	–	–	–
Platelets <100 × 10^9^/L	1.26	0.77–2.05	0.358	–	–	–
CRPH	1.32	0.80–2.17	0.277	–	–	–
MELD	1.09	0.99–1.21	0.079	–	–	–
MELD ≥ 10	1.39	0.73–2.66	0.318	–	–	–
ALBI grade ≥ 2	1.73	0.96–3.09	0.066	1.76	0.96–3.21	0.065
Total tumour diameter (cm)	1.21	0.99–1.49	0.062	1.21	0.98–1.51	0.078
Largest lesion size (cm)	1.32	0.98–1.77	0.067	–	–	–
Tumour size class (mm), ≤20 reference	–	–	–	–	–	–
21–30	1.76	1.03–3.00	0.038	–	–	–
31–50	1.64	0.77–3.47	0.199	–	–	–
Number of nodules	1.00	0.71–1.41	0.988	–	–	–
Nodule class (2 vs. 1)	0.98	0.58–1.66	0.949	–	–	–
BCLC stage A	1.33	0.77–2.31	0.311	–	–	–
AFP ≤ 20 reference	–	–	–	–	–	–
21–100	1.60	0.84–3.05	0.155	1.38	0.70–2.72	0.355
>100	2.23	1.10–4.55	0.027	2.14	1.03–4.47	0.042

OR: odds ratio CI: confidence interval; ECOG: Eastern Cooperative Oncology Group Performance Status; CCI: Charlson Comorbidity Index; BMI: body mass index; HCV: hepatitis C virus infection; HBV: hepatitis B virus infection; MASLD: metabolic dysfunction–associated steatotic liver disease; ALD: alcoholic liver disease; CRPH: clinically relevant portal hypertension; MELD: Model for End-stage Liver Disease; BCLC: Barcelona Clinic Liver Cancer; ALBI: Albumin–Bilirubin; AFP: alpha-fetoprotein.

**Table 3:** Cox regression results—survival analysis.

Variable	Univariable Analysis			Multivariable Analysis		
	HR	95% CI	*p*	HR	95% CI	*p*
Age (per 10 years)	1.24	0.99–1.53	0.055	–	–	–
Sex (male)	1.42	0.82–2.44	0.190	1.59	0.91–2.76	0.102
ECOG PST > 0 vs. 0	1.29	0.83–1.99	0.264	–	–	–
Diabetes	1.29	0.89–1.88	0.181	–	–	–
Cardiopulmonary comorbidity	1.65	1.01–2.67	0.044	1.75	1.07–2.85	0.025
CCI	1.15	1.04–1.26	0.007	–	–	–
BMI (per 10 units)	0.87	0.54–1.40	0.570	–	–	–
MASLD	1.04	0.61–1.77	0.881	–	–	–
HCV infection	1.02	0.71–1.46	0.929	–	–	–
HBV infection	0.69	0.41–1.15	0.135	–	–	–
ALD	1.20	0.81–1.76	0.362	–	–	–
Child score 6 vs. 5	1.64	1.13–2.37	0.008	1.51	1.03–2.21	0.036
Oesophageal varices	1.71	1.19–2.45	0.004	1.54	1.05–2.24	0.026
Ascites	1.40	0.82–2.37	0.231	–	–	–
Hepatic encephalopathy	2.02	0.89–4.60	0.096	–	–	–
Platelets <100 × 10^9^/L	1.50	1.05–2.15	0.027	–	–	–
CRPH	1.67	1.14–2.43	0.007	–	–	–
MELD	1.09	1.02–1.16	0.012	–	–	–
MELD ≥ 10	1.58	1.00–2.50	0.049	–	–	–
ALBI grade ≥ 2	1.25	0.83–1.88	0.293	–	–	–
Total tumour diameter (cm)	1.27	0.97–1.31	0.112	–	–	–
Largest lesion size (cm)	1.30	1.04–1.61	0.023	1.39	1.10–1.75	0.006
Tumour size class (mm), ≤20 reference	–	–	–	–	–	–
21–30	1.54	1.04–2.27	0.030	–	–	–
31–50	1.55	0.89–2.70	0.117	–	–	–
Number of nodules	0.92	0.71–1.19	0.528	–	–	–
Nodule class (2 vs. 1)	0.88	0.60–1.29	0.502	–	–	–
BCLC stage A	1.18	0.79–1.77	0.408	–	–	–
AFP ≤ 20 reference	–	–	–	–	–	–
21–100	1.75	1.11–2.75	0.016	1.52	0.96–2.41	0.073
>100	2.40	1.46–3.95	0.001	2.68	1.62–4.44	<0.001

HR: hazard ratio; CI: confidence interval; ECOG: Eastern Cooperative Oncology Group Performance Status; CCI: Charlson Comorbidity Index; BMI: body mass index; HCV: hepatitis C virus infection; HBV: hepatitis B virus infection; MASLD: metabolic dysfunction–associated steatotic liver disease; ALD: alcoholic liver disease; CRPH: clinically relevant portal hypertension; MELD: Model for End-stage Liver Disease; BCLC: Barcelona Clinic Liver Cancer; ALBI: Albumin–Bilirubin; AFP: alpha-fetoprotein.

## Data Availability

The data presented in this study are available on request from the corresponding author. The data are not publicly available due to privacy restrictions according to Italian law.

## Abbreviations

The following abbreviations are used in this manuscript:

LT	Liver Transplantation
HCC	hepatocellular carcinoma
DCD	donation after circulatory death
LDLT	living donor liver transplantation
CRLM	colorectal liver metastases
NET	neuroendocrine tumour
SLT	salvage liver transplantation
PLT	primary liver transplantation
AFP	alpha-fetoprotein
P-MWA	percutaneous microwave ablation
L-MWA	laparoscopic microwave ablation
BMI	body mass index
DAA	direct-acting antiviral
HCV	hepatitis C Virus
HBV	hepatitis B Virus
MAFLD	metabolic-associated fatty liver disease
ALBI	Albumin-Bilirubin
MELD	Model for End-stage Liver Disease
BCLC	Barcelona Clinic Liver Cancer
CT	computed tomography
TACE	transarterial chemoembolization
ST	systemic therapy
ECOG	Eastern Cooperative Oncology Group
OR	odds ratio
CI	confidence interval
IQR	interquartile range
OS	overall survival

## Appendix A

The following table (Table A1) presents the selection criteria for laparoscopic microwave ablation (L-MWA) in hepatocellular carcinoma (HCC) patients at Padua University Hospital. These criteria include ineligibility for liver resection (LR) and factors that make patients unsuitable for percutaneous ablation, such as tumour location, liver function, coagulopathy, and ultrasound visualisation issues.

**Table A1:** Selection criteria for laparoscopic microwave ablation in hepatocellular carcinoma (HCC) patients at Padua University Hospital.

**Ineligibility for liver resection** (severe portal hypertension, location, impaired liver function)
**Unsuitability for percutaneous ablation due to:**
Critical location (superficial nodules, caudate lobe, superoposterior segments, adjacent the biliary tree or major hepatic vessels) Proximity to colon, duodenum, gallbladder, diaphragm, heart Severe coagulopathy (prothrombin time <40%, platelets count <30 × 10^9^ Untreatable ascites Ultrasound visualization issues (obesity, previous surgery)

These criteria have been previously described by Lanari et al. [38].
